# Supplementary material for: WordCommentsAnalyzer: A windows software tool for qualitative research
Source: F1000Res. 2018 Sep 4;7:536. Originally published 2018 May 3. [Version 2] doi: 10.12688/f1000research.14819.2 (PMC6137411; doi:10.12688/f1000research.14819.2)
Supplement: Supplementary file 3 [file f1000research-7-17614-s0002.tgz › b2a79496-4ada-47c4-9682-a99ce867836d.zip!/Eight Iranian Health Journals/Yafteh _9.docx]

© Yafteh  . This work is licensed under CC BY 4.0 (https://creativecommons.org/licenses/by/4.0/).

Volume & Issue: دوره 18 - شماره 1

Title: بررسی سلامت آب شرب شهرستان نورآباد لرستان از نظر پتانسیل خوردگی و رسوب‌گذاری با استفاده از اندیس‌های خوردگی در سال 1392

http://yafte.lums.ac.ir/article-1-2210-fa.html

مقدمه: خوردگی می‌تواند باعث آسیب‌های اقتصادی، کاهش عمر مفید تأسیسات آبرسانی و بیماری در مصرف کنندگان گردد. لذا هدف از این تحقیق، بررسی پتانسیل خورندگی و رسوب گذاری منابع آب شرب شهرستان نورآباد لرستان می‌باشد. مواد و روش ها: این مطالعه از نوع توصیفی مقطعی بوده که طی فصول پاییز و زمستان از 7‌حلقه چاه و 2‌مخزن تأمین آب شرب شهرستان نورآباد نمونه برداری صورت گرفت. بدین منظور پارامترهای pHs،pH ، درجه حرارت، سختی کلسیم، قلیاییت کل و TDS اندازه‌گیری و بر اساس مقدار آن‌ها، چهار اندیس خوردگی شامل لانژلیه، رایزنر، تهاجمی و پوکوریوس تعیین شدند. یافته ها:  نتایج این بررسی نشان داد که شاخص لانژلیه، رایزنر، پوکوریوس و تهاجمی در فصول مورد بررسی به ترتیب در محدوده 0/07+ تا 0/61+، 7/44 تا 8/06، 6/9 تا 7/25 و 11/38 تا 11/9 می‌باشد. بر اساس شاخص‌های رایزنر، پوکوریوس و تهاجمی تمامی منابع تأمین آب شرب شهرستان نورآباد خورنده بوده به‌طوری‌که بر اساس شاخص لانژلیه همه منابع تأمین آب شرب شهرستان نورآباد از نوع رسوب‌گذار بود. بحث و نتیجه گیری: بررسی شاخص‌های مربوطه نشان می‌دهد که آب آشامیدنی شهر نورآباد خورنده بوده و لذا بایستی کیفیت آب موجود در شبکه توزیع به‌طور مستمر مورد پایش قرار گرفته و اقدامات لازم در زمینه کنترل خوردگی اعمال نمود. بهترین راهکارهای قابل اجرا در مورد سیستم آبرسانی شهر نورآباد برای کاهش خورندگی آب، کنترل مداوم pH و مکانیسم کلرزنی و همچنین استفاده از لوله‌ها و تأسیسات مقاوم به خورندگی می‌باشند.

Title: تعیین کمی بار ویروسی هپاتیت C با استفاده از روش Real-Time PCR In-House در بیماران آلوده به هپاتیت C در شهرستان خرم آباد

http://yafte.lums.ac.ir/article-1-2211-fa.html

مقدمه: روش‌های تشخیصی مولکولی ابزار عمده‌ای در مدیریت بیماران مبتلا به ویروس هپاتیتHCV)C  هستند. مطالعات نشان داده‌اند که بار ویروسی با مرحله عفونت و میزان پاسخ به درمان مرتبط می‌باشد و لذا ارزیابی و تعیین بار ویروسی اهمیت بسزایی دارد. هدف از این مطالعه بکارگیری روش دقیق و در عین حال ارزان قیمت جهت سنجش کمی بار ویروسی در پلاسمای بیماران می‌باشد. مواد و روش ها: پس از راه اندازی و تعیین اعتبار روش راه اندازی شده، سنجش کمی بار ویروسی بر روی پلاسمای 200 بیمار آلوده به فرم مزمن HCV انجام شد. بار ویروس با استفاده از منحنی استاندارد خارجی و با بهره گیری از پانل استاندارد RNA صورت گرفت. نتایج حاصل از این روش با نتایج بدست آمده از کیت تجاری آرتوس مقایسه شد. یافته ها:  محدوده تعیین این روش IU/ml 50 می‌باشد. میانگین بار ویروسی اندازه‌گیری شده در مقیاس لگاریتمی (22/0 ± 81/5)، (05/0P<) بدست آمد. آنالیز موازی نمونه‌ها با استفاده از این روش و کیت تجاری آرتوس همبستگی خوبی بین دو روش نشان داد (05/0>P‌، 988/0=R2). بحث و نتیجه گیری: تعیین کمی بار ویروسی HCV در افراد آلوده به این ویروس در شهرستان خرم آباد برای اولین بار گزارش شده است. توجه به نتایج، این روش دارای حساسیت خوب و ویژگی برای سنجش کمی HCV RNA در مقیاس بزرگ است. این روش می‌تواند جایگزین مناسبی برای کیت‌های تجاری به‌ویژه برای ارزیابی بالینی درمان باشد.

Title: بررسی موانع پژوهش از دیدگاه اساتید دانشگاه علوم پزشکی لرستان و ارتباط آن با عملکرد پژوهشی ایشان

http://yafte.lums.ac.ir/article-1-2212-fa.html

مقدمه: شناسایی نقاط ضعف پژوهش اولین گام برای سامان بخشیدن به پژوهش در جامعه است. چون عمده فعالیت‌های پژوهشی توسط اعضای هیأت علمی دانشگاه‌ها صورت می‌گیرد در این پژوهش سعی شده تا ارتباط موانع پژوهش از دیدگاه اعضای هیأت علمی دانشگاه علوم پزشکی لرستان با عملکرد پژوهشی ایشان بررسی گردد. مواد و روش ها: این مطالعه مقطعی بر روی اساتید دانشگاه علوم پزشکی لرستان به روش سرشماری انجام گردید. یک پرسشنامه محقق ساخته برای ارزیابی متغیرهای جمعیت شناختی و آگاهی و نگرش اعضای هیأت علمی نسبت به موانع موجود در مراحل مختلف پژوهش تدوین و به صورت خود ایفا تکمیل شد و ارتباط آن با نمرات ارزشیابی سالیانه اساتید در معیار پژوهشی با استفاده از آزمون مجذور کای، من-ویتنی و کروسکال-والیس بررسی گردید. یافته ها:  متغیرهای دانشکده محل خدمت، کمیت تدریس، انگیزه پژوهشی اولیه، آگاهی نسبت به متغیرهای اصول روش تحقیق، اصول جستجو در بانک‌های اطلاعاتی پزشکی، اصول پروپوزال نویسی و اصول مقاله نویسی و نیز نگرش نسبت به متغیرهای اولویت‌های پژوهشی مصوب، نگارش پروپوزال، کیفیت مشاوره پژوهشی، فرآیند تصویب طرح‌ها در شوراهای پژوهشی، تسهیلات پژوهشی، نگارش مقاله، فرآیند داوری مقالات داخلی، ارائه مقاله در همایش‌ها و مشارکت در پایان نامه‌ها در ارتباط معنادار با عملکرد پژوهشی افراد بودند (005/0P<). بحث و نتیجه گیری: به نظر می‌رسد با اعطای امتیازات ویژه به جمعیت‌های خاص نظیر اعضای هیأت علمی دانشکده پزشکی، افراد دارای کمیت تدریس بالا و افراد دارای آگاهی و نگرش نامطلوب بتوان انگیزه ایشان جهت انجام فعالیت‌های پژوهشی را تقویت کرد.

Title: بررسی میزان شیوع پیتریازیس ورسیکالر در خوابگاه‌های دانشگاه علوم پزشکی لرستان به سه روش تراشیدن پوست، استفاده از چسب اسکاچ و لامپ وود

http://yafte.lums.ac.ir/article-1-2213-fa.html

مقدمه: پیتریازیس ورسیکالر عفونت مزمن، عود کننده و معمولاً بدون علامت لایه شاخی پوست می‌باشد که به‌وسیله‌ی مخمرهای لیپوفیل از جنس مالاسزیا ایجاد می‌شود. انتقال انسان به انسان از طریق تماس مستقیم امکان پذیر است. مطالعه حاضر با هدف بررسی میزان شیوع پیتریازیس ورسیکالر در دانشجویان خوابگاه‌های دانشگاه علوم پزشکی لرستان به‌ویژه افراد با ضایعات مشکوک به پیتریازیس ورسیکالر به سه روش تراشیدن پوست، استفاده از چسب اسکاچ و لامپ وود انجام گردید. مواد و روش­ ها: در این مطالعه توصیفی- مقطعی، 115 نفر از دانشجویان خوابگاه‌های دانشگاه علوم پزشکی لرستان به سه روش تراشیدن پوست، استفاده از چسب اسکاچ و لامپ وود مورد مطالعه قرار گرفتند. یافته­ ها:  از بین 115 نفر، 66/94 درصد دختر و 33/05 درصد پسر بودند. میانگین سنی افراد 21/38 سال بود. 25 درصد دانشجویان پسر و 11/1 درصد دانشجویان دختر مبتلا بودند. شیوع پیتریازیس ورسیکالر به هر سه روش فوق 7/15 درصد تعیین شد. در مطالعه حاضر، شایع‌ترین محل‌های ابتلا، کتف و گردن بود. بحث و نتیجه ­گیری: مطالعه اخیر شیوع بالایی از پیتریازیس ورسیکالر را در دانشجویان دانشگاه علوم پزشکی لرستان نشان داد. شیوع پیتریازیس ورسیکالر بین دختران و پسران تفاوت معناداری داشت و در پسران شایع‌تر بود. در مقایسه دو به دویی نتایج بدست آمده از هر سه روش با استفاده از ضریب کاپا، روش استفاده از چسب اسکاچ بیشترین تطابق نتیجه را با دو روش دیگر داشت.

Title: آلودگی انتروباکتریاسه در آب آشامیدنی چاه‌های شهرستان رومشکان

http://yafte.lums.ac.ir/article-1-2214-fa.html

مقدمه: آب یک مایع حیاتی است که توسط عوامل متعدد بیولوژیکی مانند انگل، قارچ، ویروس و باکتری آلوده می‌شود. انتروباکتریاسه‌ها بخصوص اشرشیاکلی از مهم‌ترین شاخص‌های آلودگی آب با مدفوع می‌باشند. هدف از این مطالعه بررسی انتروباکتریاسه‌ها در آب آشامیدنی چاه‌های شهرستان رومشکان می‌باشد. مواد و روش ­ها: تعداد 160 نمونه 2/5 لیتری آب جمع آوری و در اسرع وقت و تحت شرایط مطلوب دمایی به آزمایشگاه انتقال و در آنجا با عبور از فیلترهای 0/45 میکرونی تغلیظ گردید. سپس کلنی‌های انفرادی تشکیل شده در محیط کشت مک‌کانکی، برای تعیین نوع باکتری روی دیگر محیط‌های افتراقی انتقال یافتند. یافته­ ها: تعداد 18 مورد (11/25%) از آب آشامیدنی چاه‌ها به اشرشیاکلی آلوده بودند که در اکثر موارد با سایر انتروباکتریاسه‌ها مانند انتروباکتر، سیتروباکتر و کلبسیلا توأم بود. این آلودگی‌ها با فاصله چاه‌های فاضلاب و درب‌های معیوب چاه‌ها رابطه معنی داری داشتند. بحث و نتیجه­ گیری: هر چند میزان آلودگی نسبت به بعضی مطالعات انجام شده پایین‌تر بود ولی بر اساس استاندارد WHO بسیار بالاست. پیشنهاد می‌شود پیش از آنکه بیماری‌های منتقله  از آب چاه­ها اپیدمیک شوند دست‌اندرکاران با لوله کشی و یا راه­های ممکن اقدام نمایند.

Title: تبیین نقش ارزش‌ها در اخلاق حرفه‌ای پرستاری: یک مطالعه تماتیک

http://yafte.lums.ac.ir/article-1-2215-fa.html

مقدمه: شناخت ارزش‌ها در‌ بین پرستاران به دلیل نقش اساسی که بر سلامتی و حیات انسان‌ها دارند اهمیت زیادی دارد زیرا کیفیت رفتار و عملکرد حرفه‌ای آنان را شکل می‌دهند. مطالعه حاضر با‌هدف کشف ارزش‌های مؤثر بر مراقبت اخلاق حرفه‌ای پرستاران ایرانی انجام شد. مواد و روش­ ها: تجارب 23 مشارکت‌کننده پرستار، سرپرستار، پزشک، مددجو و همراه بیمار از اسفند 1391 تا مرداد 1393 در مطالعه‌ا‌ی کیفی با رویکرد استقرایی، به روش مصاحبه عمیق نیمه ساختاریافته بررسی شد. با کسب رضایت مشارکت‌کنندگان مصاحبه‌ها ضبط، بازنویسی شده و با ‌روش تحلیل درون‌مایه‌ای براون و کلرک تجزیه‌وتحلیل شد. دقت و استحکام داده‌ها با معیارهای لینکلن و گوبا موردبررسی قرار گرفت. یافته ­ها: درنهایت یک درون‌مایه سایه‌بانی با عنوان باورهای مؤثر بر مراقبت اخلاق حرفه‌ای پرستاری شکل گرفت که حاصل تلفیق دو درون‌مایه فرعی بود. درون‌مایه‌ فرعی ارزش‌های عام را پنج درون‌مایه اولیه (عقیده به کرامت ذاتی مددجو، عاطفه و علاقه به انسان‌ها، احساس نگرش و رفتار با بیمار مانند اعضای خانواده خود، باورهای اخلاقی معنوی مؤثر بر مراقبت و وجدان و تعهد حرفه‌ای پرستار) و درون‌مایه فرعی ارزش‌های خاص را سه درون‌مایه‌ اولیه (شناخت حفظ و حمایت از حقوق بیمار، تنگناهای اخلاقی موجود در حرفه، پرهیز از روابط غیرحرفه‌ای و سوء‌استفاده از مددجو) پدید آوردند. بحث و نتیجه­ گیری: نتایج با مطالعات مشابه داخل و خارج کشور همخوانی‌های کلی داشت. پیشنهاد می‌شود برای عملی شدن ارزش‌ها در مراقبت‌های پرستاری در بالین سازوکارهای سنجش و نظارت مداوم فراهم گردد.

Title: مقایسه سیستم‌های مغزی رفتاری بیماران مبتلا به مولتیپل اسکلروزیس و افراد عادی

http://yafte.lums.ac.ir/article-1-2216-fa.html

مقدمه: هدف پژوهش حاضر مقایسه سیستم‌های مغزی رفتاری افراد دارای بیماری مولتیپل اسکلروزیس (ام اس) و افرادی عادی بوده است. مواد و روش­ ها: روش پژوهش حاضر از نوع علی مقایسه‌ای بوده است. جامعه آماری شامل تمام افراد عادی و تمام بیماران مبتلا به ام اس می‌باشد که در تابستان و پاییز سال 1393 به متخصصین مغز و اعصاب در استان لرستان مراجعه نموده‌اند. از جامعه آماری گروه نمونه‌ای به‌اندازه 117 نفر (75 فرد مبتلا و 42 فرد عادی) با استفاده از روش نمونه گیری در دسترس انتخاب شدند، سپس پرسش نامه شخصیتی گری- ویلسون بر روی آنان اجرا شد. برای تجزیه و تحلیل داده‌ها از روش تحلیل واریانس چند متغیری (مانوا) استفاده شد. یافته­ ها: یافته‌ها نشان داد، در خرده مقیاس فعال‌ساز رفتاری، افراد مبتلا به ام اس به‌طور معناداری نمرات پایین‌تری را در مقایسه با افراد عادی کسب کردند. بحث و نتیجه ­گیری: آنچه از یافته‌ها بر می‌آید این است که نمره پایین در فعال‌سازی رفتاری به‌عنوان یکی از عوامل آسیب شناختی در بیماری‌های مزمن از جمله ام اس مطرح می‌باشد و نیازمند درمان روان شناختی است.

Title: بررسی غلظت خونی وانادیوم و کروم در بیماران سندروم متابولیک با و بدون دیابت تیپ 2

http://yafte.lums.ac.ir/article-1-2217-fa.html

مقدمه: بیماری دیابت ششمین علت مرگ و میر می‌باشد و امروزه تلاش‌های زیادی برای شناسایی عوامل تأثیر گذار آن صورت می‌گیرد این مطالعه با هدف بررسی ارتباط بین غلظت خونی کروم و وانادیوم با بیماری سندروم متابولیک با و بدون دیابت تیپ 2 می‌باشد. مواد و روش­ ها: در این مطالعه ابتدا نمونه‌های خونی توسط اسید نیتریک هضم شد. غلظت خونی کروم و وانادیوم در گروه سندروم متابولیک با دیابت تیپ 2، 47 بیمار (30 زن و 17 مرد) و گروه سندروم متابولیک بدون دیابت تیپ 2، 45 بیمار (33 زن و 12 مرد) و گروه شاهد 35 نفر (23 زن و 12 مرد) با استفاده از دستگاه جذب اتمی بدون شعله اندازه گیری شد. یافته­ ها: نتایج حاصل از اندازه گیری نشان داد اختلاف معناداری بین گروه‌ها وجود دارد (05/0>P). میزان عناصر اندازه‌گیری شده در گروه نرمال بیشتر از گروه سندروم متابولیک بدون دیابت تیپ 2 و در گروه سندروم متابولیک بدون دیابت تیپ 2 بیشتر از گروه سندروم متابولیک با دیابت تیپ 2 می‌باشد. بحث و نتیجه ­گیری: نتایج حاصل از مطالعه ما نشان می‌دهد سطح خونی عناصر اندزه‌گیری شده با بیماری دیابت و سندروم متابولیک ارتباط دارد. در نتیجه با انداز گیری سطح این عناصر و دادن آن‌ها به‌صورت مکمل از بروز و یا پیشرفت بیماری می‌توان جلوگیری کرد.

Title: بررسی ارتباط جراحی رحمی قبلی با میزان بروز آدنومیوز

http://yafte.lums.ac.ir/article-1-2218-fa.html

مقدمه: آدنومیوز یکی از اختلالات شایع خوش‌خیم در میان زنان سنین پری منوپوزال است. این بیماری می‌تواند بدون علامت باشد یا با علائمی چون بزرگی رحم، درد لگنی، خونریزی‌های زیاد قاعدگی و دیس منوره ظاهر شود. هدف اصلی این مطالعه بررسی سابقه جراحی قبلی به عنوان یک ریسک فاکتور برای آدنومیوز بود. مواد و روش ­ها: این مطالعه توصیفی مقطعی به بررسی زنانی پرداخت که طی سال‌های 1385- 1380 در بیمارستان شریعتی بندرعباس تحت هیسترکتومی غیر اورژانسی قرار گرفته بودند. اطلاعات شامل سن، تعداد حاملگی و زایمان، اندازه رحم، سابقه سقط خود به خود، سابقه جراحی قبلی رحمی (سزارین و کورتاژ) و میومکتومی از پرونده‌های پزشکی افراد و وجود آدنومیوز نیز از دفاتر ثبت پاتولوژی جمع آوری گردید. جهت تجزیه و تحلیل آماری از نرم افزار SPSS 16 و آزمون‌های آماری T و ANOVA استفاده شد. 05/0>P معنی دار در نظر گرفته شد یافته­ ها: از 191 زن مورد مطالعه، 72 نفر (7/37 درصد) پاتولوژی مثبت داشتند. شیوع آدنومیوز در این مطالعه 7/37 درصد بود. زنان مبتلا به آدنومیوز به‌طور واضح سابقه قبلی جراحی رحمی بیشتری داشتند (03/0=P). ولی سن، منارک، سابقه سقط، سایز رحم، تعداد حاملگی و بارداری در هر دو گروه تفاوت معنی داری نداشتند. بحث و نتیجه­ گیری: این مطالعه نشان داد که سابقه جراحی قبلی رحمی یک ریسک فاکتور برای آدنومیوز است. با کاهش جراحی‌های رحمی غیرضروری می‌توان از ایجاد آدنومیوز و عوارض ناشی از آن کاست.

Title: بررسی اثرات فلاونوئید کوئرستین بر بیماری کبد چرب غیر الکلی

http://yafte.lums.ac.ir/article-1-2219-fa.html

مقدمه: شیوع بیماری کبد چرب غیر الکلی به‌طور جهانی افزایش یافته و در جمعیت عمومی جهان 24-10% می‌باشد. مقاومت به انسولین و تجمع چربی در سلول‌های کبدی در پاتوژنز این بیماری نقش مؤثری دارند. اخیراً استفاده از آنتی‌اکسیدان‌ها در پیشگیری و درمان بیماری‌ها مورد توجه قرار گرفته است. کوئرستین، آنتی‌اکسیدانی از گروه فلاونوئیدهاست. مطالعه مروری حاضر با هدف بررسی اثر کوئرستین بر بیماری کبد چرب غیر الکلی انجام گرفته است. مواد و روش ­ها: با استفاده از کلید واژه‌های NAFLD، antioxidant، quercetin، insulin resistance، liver activity، lipid‌و inflammation. جستجو در پایگاه‌های اینترنتی Pubmed، Sciencedirect، Scopus وgoogle scholar صورت گرفت. در نهایت از بین مقالات جستجو شده، 20 مقاله کامل و 12 چکیده که مطابقت موضوعی با هدف مطالعه حاضر داشتند، بررسی شد. یافته‌ها: کوئرستین علاوه بر خواص ذاتی آنتی‌اکسیدانی، ویژگی‌هایی در زمینه کاهش معنی دار التهاب، کاهش سطح چربی‌های سرمی و تجمع چربی کبدی، کاهش مقاومت به انسولین از طریق افزایش فعالیت آنزیم هگزوکیناز، افزایش محتوای گلیکوژن عضلانی و کبدی، افزایش فسفریلاسیون و فعال سازی گیرنده‌های انسولینی، ارتقای کارکرد کبدی و کاهش آنزیم‌های ترانس آمیناز کبدی سرم و جلوگیری از تولید فاکتورهای فیبروزه کننده کبد را نیز دارا می‌باشد. بحث و نتیجه گیری: با توجه به اثرات آنتی‌اکسیدانی، ضد التهابی، هیپولیپیدمیک، افزایش حساسیت به انسولین و ضد فیبروزی کوئرستین در مطالعات سلولی و حیوانی، استفاده از آن ممکن است سبب پیشگیری و بهبود کبد چرب غیر الکلی در انسان نیز بشود.

Title: بررسی اثربخشی نوروفیدبک همراه با تمرین‌های شناختی رایانه‌ای در بهبود حافظه فعال در بزرگسالان دارای اختلال نقص توجه/بیش فعالی

http://yafte.lums.ac.ir/article-1-2220-fa.html

مقدمه: اختلال نقص توجه/بیش فعالی، یکی از اختلالات روان‌شناختی شایع در افراد می­باشد که از کودکی تا بزرگسالی تداوم می‌یابد و در ابعاد مختلفی از زندگی فرد، نظیر زندگی شخصی، اجتماعی، حرفه­ای، کارکرد اجرایی نظیر حافظه فعال اختلال ایجاد می­کند. مطالعات متعددی نشان دهنده ارتباط نزدیک نقایص حافظه فعال و اختلال نقص توجه/بیش فعالی بوده است. با توجه به فقدان مطالعه در خصوص اثربخشی نوروفیدبک در بهبود حافظه فعال در بزرگسالان دارای اختلال، این مطالعه با هدف بررسی اثربخشی نوروفیدبک در حافظه فعال شکل گرفته است. مواد و روش ­ها: طرح پژوهش از نوع شبه آزمایشی، با پیش آزمون-پس آزمون و گروه کنترل می­باشد. جامعه آماری را بزرگسالان دارای اختلال نقص توجه/بیش فعالی که به کلینیک آتیه در تهران مراجعه می­کنند، تشکیل دادند؛ که از بین آن‌ها پس از بررسی ملاک­های ورود و خروج، دو گروه آزمایشی و کنترل و در هر گروه 8 آزمودنی، به‌طور تصادفی جایگزین گردیدند. ابزارهای پژوهش عبارت‌اند از پرسشنامه اضطراب بک، افسردگی بک، آزمون عملکرد پیوسته دیداری شنیداری رایانه­ای و آزمون علائم حیاتی سیستم عصبی مرکزی. اطلاعات، از طریق SPSS و با استفاده از روش آماری یومن ویتنی مورد تجزیه و تحلیل قرار گرفت. همچنین از آزمون‌های تی مستقل، ویلکاکسون و کروسکال والیس نیز جهت نتایج تکمیلی استفاده شد. در این مطالعه پروتکل مورد استفاده، تقویت موج بتا در نقطهFZ  می‌باشد. یافته ­ها:  نتایج به دست آمده نشان داد که نوروفیدبک منجر به افزایش معنادار ظرفیت حافظه فعال در بزرگسالان دارای اختلال نقص توجه/بیش فعالی گردید. بحث و نتیجه ­گیری: با توجه به نتایج به دست آمده در این پژوهش که همسو با نتایج بسیاری از پژوهش‌های صورت گرفته در این زمینه می‌باشد، نوروفیدبک، موجب افزایش فعالیت لوب فرونتال و همچنین فعال سازی مدارهای عصبی مربوط به کارکردهای اجرایی و حافظه فعال گردیده و موجب بهبود نقایص کارکردهای اجرایی و حافظه فعال در افراد دارای اختلال نقص توجه/بیش فعالی می‌گردد. در نتیجه، با توجه به اثر بخشی نوروفیدبک در بهبود حافظه فعال، استفاده از این روش برای درمان‌های مشابه پیشنهاد می‌گردد.

Title: اثر ضد ویروسی سه گونه گیاهی ایران بر روی ویروس هرپس سیمپلکس تیپ یک

http://yafte.lums.ac.ir/article-1-2221-fa.html

مقدمه: گیاهان در زندگی انسان دارای جایگاه ویژه ای بوده اند و کاربرد دارویی آنها در دست نوشته های بسیاری از دانشمندان جهان آمده است. امروزه درمان بیماری های ویروس هرپس سیمپلکس تیپ یک با داروهای شیمیایی به دلیل پیدایش مقاومت دارویی و دوره نهفتگی ویروس با چالش هایی روبرو شده است، پس نیاز به داروهای ضد هرپسی نوین می باشد. در این پژوهش اثر ضد ویروسی چای سبز، گل گاوزبان و خرزهره با داشتن پیشینه درمانی در پزشکی سنتی بر روی ویروس هرپس سیمپلکس تیپ یک بررسی شد. مواد و روش ­ها: نخست عصاره آبی گیاهان با روش جوشاندن فراهم گردید و پس از سنجش سیتوکسیسیته آنها بر روی دودمان یاخته ایHep-2 (Human epithelial type 2) با ارزیابی C‏PE (cytopathic effect) ، اثر ضد هرپسی عصاره های گیاهی با روش بازدارندگی ازCPE  ویروس بررسی گردید. یافته­ ها:  عصاره خرزهره بیشترین توکسیسیته را روی یاخته های Hep-2 داشت (غلظتهای بالای ml/μg 50) و گیاه چای سبز بیشترین ویژگی بازدارندگی از تکثیر ویروس را نشان داد. گل گاوزبان اثر ضد هرپسی کمتری داشت. چای سبز در دامنه غلظتی ml/μg 50-1000 و گل گاوزبان در غلظت های بالای μg/ml 300 بازدارنده تکثیر کامل ویروس بودند. بحث و نتیجه­ گیری: چای سبز و گل گاوزبان در غلظت های غیرتوکسیک به خوبی توانستند از تکثیر کامل ویروس هرپس سیمپلکس تیپ یک پیشگیری کنند. برای پیداکردن مکانیسم اثر این داروها نیاز به پژوهش های بیشتر می باشد تا بتوان آنها را در ساخت داروهای ضدهرپسی نوین به کار برد.
